# Biomimetic Ultrasonic Vibrator with Broadband Characteristics Inspired by Leaf-Cutting Ants

**DOI:** 10.3390/biomimetics9040247

**Published:** 2024-04-19

**Authors:** Wenshuai Wu, Guang Yao, Mingshuo Zhang, Xinggang Jiang, Deyuan Zhang

**Affiliations:** 1 School of Mechanical Engineering and Automation, Beihang University, Beijing 100191, China; wu_ws@buaa.edu.cn (W.W.); yghit316@163.com (G.Y.); sy2107417@buaa.edu.cn (M.Z.); sdjxg@163.com (X.J.); 2 Institute of Bionic and Micro-Nano Systems, Beihang University, Beijing 100191, China

**Keywords:** power ultrasound, leaf-cutting ant, broadband characteristics, load-carrying capacity, minimally invasive surgical instruments

## Abstract

Power ultrasound is widely used in industrial production, medical equipment, aerospace, and other fields. Currently, there are two main types of commonly used power generation devices: piezoelectric ultrasonic transducers and magnetostrictive ultrasonic transducers. However, in certain situations with limited external dimensions, the applications of existing power ultrasound devices are limited. In nature, leaf-cutting ants excite vibrations through their tiny organs. Inspired by the vibratory organs of leaf-cutting ants, a new type of biomimetic ultrasonic vibrator (BUV) comprising a scraper, dentate disc, and fixture system was proposed, fabricated, and tested in this study. The experimental results showed that the BUV could operate in the frequency range of 16.8–19 kHz. Within the working frequency range, the vibration of the BUV was stable and the amplitude of the vibration displacement was greater than 22 µm. The operating frequency band of the BUV was broader than those of the piezoelectric and magnetostrictive ultrasonic transducers. In addition, the BUV can cut soft rubber and pig tissues with sufficient output power and load-carrying capacity. The BUV, as a new type of power ultrasonic excitation device, is expected to be applied in high-power micro operating scenarios, such as minimally invasive surgical instruments.

## 1. Introduction

Power ultrasound is widely used in aerospace, healthcare, and industrial production [1,2]. Generally, the vibration frequency of a power ultrasound is between 16 and 100 kHz. Owing to its high power density, power ultrasound can generate strong forces and thermal effects on the target object [2]. For example, ultrasonic cleaning can efficiently remove firmly adhered stains by utilizing the cavitation effect, and can also achieve cleaning of complex parts efficiently [3]. Ultrasonic machining can be used to process extremely hard materials, thereby ensuring high machining accuracy and surface integrity [4,5,6]. Ultrasound-assisted machining can reduce the cutting force and prolong tool life, achieving efficient machining of difficult-to-process materials in the aerospace field [7,8,9,10]. Ultrasonic scalpels can achieve the minimally invasive cutting of body tissues, reduce bleeding, and ensure rapid wound healing [11,12,13]. An ultrasonic osteotomy knife can achieve minimally invasive resection of bone tissue. High-performance ultrasound generation devices are necessary to achieve the application of power ultrasound in the above scenarios and obtain excellent results.

Currently, piezoelectric and magnetostrictive ultrasonic transducers are the two most commonly used power ultrasound generation devices [14,15,16,17]. Piezoelectric ultrasonic transducers have a simple structure and high power density and are widely used in mechanical processing, medical and health care, food processing, microelectronics, and ultrasonic cleaning [1]. Currently, magnetostrictive ultrasonic transducers primarily use the giant magnetostrictive effect to convert energy into ultrasonic vibrations. Giant magnetostrictive materials have high power densities, large magnetostrictive coefficients, and fast response speeds [1,18]. In recent years, magnetostrictive transducers have attracted the attention of researchers and have been widely used in cutting processing, ultrasonic welding, and other fields. Piezoelectric and magnetostrictive ultrasonic transducers are the most widely used ultrasonic transducers. However, these two types of ultrasonic transducers are generally relatively large, making them difficult to apply in situations where space size is limited. For example, in the field of minimally invasive surgery, it is difficult to combine piezoelectric ultrasonic transducers with flexible surgical robotic arms due to their size.

To solve these problems, Lockhart et al. proposed a micropiezoelectric ultrasonic transducer using single-crystal silicon as the base material [19]. The new transducer is approximately 80 mm long and 10 mm wide. It has a small overall volume and achieves good vibrational effects. However, because silicon materials break down easily, they cannot be used in clinical medicine. Martin et al. further proposed a microultrasonic transducer using a titanium alloy as the base material [20]. This type of transducer exhibits good working performance, and its characteristic size is approximately 22 mm. However, it cannot fully meet the application requirements in the minimally invasive medical field.

A type of mechanical exciter is used in the field of vibration excitation, which typically uses mechanical mechanisms to achieve vibration excitation, such as a cam mechanism [21] or a linkage mechanism [22]. This type of exciter is primarily used to generate vibrations at frequencies below 100 Hz; there are almost no applications that can generate ultrasonic vibrations. Savart used a gear device called the Savart wheel to excite ultrasonic vibrations [23]; however, this device was mainly used to generate a standard sound of accurate frequency and was a sound frequency calibration instrument. However, its feasibility as a power ultrasound device has not yet been explored. Relevant research has shown that some insects have specific excitation organs [24,25,26,27,28]. These excitation organs achieve vibrational excitation through mechanical structures; however, they usually produce high-frequency vibrations. The structures of these excitation organs are small; however, they are capable of producing high-power vibrations. For instance, in leaf-cutting ants, due to physical limitations, their excitation organs are less than 100 µm in size. However, the vibrations generated by these excitation organs can help the teeth complete the leaf-cutting process, effectively reducing the biting force required for cutting [25]. Therefore, a new type of high-power mechanical ultrasonic vibrator could be designed by imitating the excitation organs of leaf-cutting ants, providing a new solution for achieving micropower ultrasonic excitation.

In this study, a biomimetic mechanical ultrasonic excitation device was designed by observing and characterizing the excitation organs of leaf-cutting ants. A structure combining a dentate disc and scraper was proposed to mimic the spatial pose relationship and size ratio of various components of the excitation organs of leaf-cutting ants. The remainder of this paper is organized as follows: First, a brief introduction is provided to the structures of the excitation organs of the leaf-cutting ant, and the structural design, working mode, and functions of each part of the biomimetic mechanical vibrator are explained. Subsequently, a vibration model of the device was established, and the working characteristics were analyzed based on this vibration model. The nonlinear factor of the biomimetic excitation system was identified, and the broadband characteristics caused by this nonlinear factor were illustrated. Then, prototypes of the biomimetic vibrator were created, an experimental testing platform was built, and the corresponding finite element calculation models were established. The vibration output of the prototype biomimetic vibrator was experimentally measured and simulated, and the working frequency band of the device was tested. Finally, cutting experiments on biological tissues and soft rubber materials are conducted to test the output power and load-carrying capacity of the vibrator.

## 2. Excitation Organs of Leaf Cutting Ant and the Biomimetic Ultrasonic Vibrator (BUV)

### 2.1. Excitation Organs of Leaf Cutting Ant

**Figure 1:**
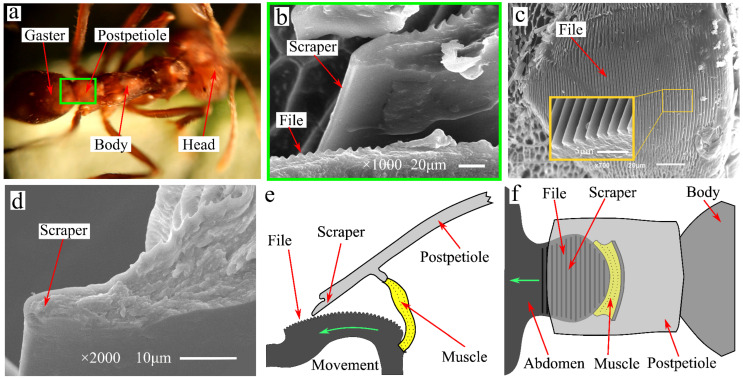
Leaf cutting ant and excitation organs. (**a**) *Atta cephalotes*, (**b**) excitation organs of leaf-cutting ant, (**c**) the file and teeth, (**d**) the tip of the scraper, (**e**,**f**) Structure diagram of excitation organs.

The leaf-cutting ant (*Atta cephalotes*) native to South America cuts a large number of plant leaves and uses the resulting leaf fragments as raw materials for food cultivation. During the process of cutting plant leaves, leaf-cutting ants use their own excitation organs to generate vibrations. As shown in Figure 1a, the excitation organs of the leaf-cutting ant are located at the junction of the gaster and postpetiole. The excitation organs include three parts: the file, scraper, and driving muscles, as shown in Figure 1b. The file is located at the forepart of the gaster, and the scraper is at the rear edge of the postpetiole. The file area contained a large amount of micro teeth, as shown in Figure 1c. The external dimensions of the micro tooth are relatively uniform, with a tooth pitch of approximately 3 µm and an approximately sinusoidal tooth profile. As shown in Figure 1d, the tip of the scraper is arc-shaped, with a diameter of approximately 6 µm. The muscles connect the postpetiole to the gaster, as shown in Figure 1e,f. During the operation of the excitation organ, the muscles drive the gaster movement relative to the postpetiole, causing the scraper to contact the file surface and slide along the sound file surface. During the relative motion, the teeth on the surface of the file come into contact with the scraper tip, and the contact force between these two parts forces the scraper tip to produce a displacement perpendicular to the sliding direction. Under the drive of the file teeth, the tip of the scraper moves away from the sound file body, and the back plate is forced to undergo elastic deformation. After the scraper slides past the highest point of the tooth in contact, due to the recovery of its own elastic deformation, the scraper tip produces displacement near the direction of the file body. The scraper slides continuously along the surface of the file and sequentially contacts the teeth of the file. The periodic reciprocating displacement generated by the scraper tip excites the postpetiole to form an elastic vibration. The vibrations generated by the excitation organs can be transmitted to the teeth of the head of the leaf-cutting ant through the postpetiole and thorax. Tautz et al. showed that vibration can reduce cutting resistance during leaf cutting, which is beneficial for the smooth progress of the cutting process [25]. Yao et al. found that the main frequency of vibration generated by the excitation organs of leaf-cutting ants was approximately 1 kHz [27]. After transmission by scrapers and the body, the main frequency of the head vibration of leaf-cutting ants was approximately 1.67 kHz [27]. The characteristic size of the excitation organs of the leaf-cutting ant is approximately 0.2 mm, but it can generate sufficient power of vibration to assist teeth in cutting plant tissue. The vibration excitation phenomenon of the leaf-cutting ants inspired us to design a BUV and conduct performance testing.

### 2.2. Design of Biomimetic Vibrator

**Figure 2:**
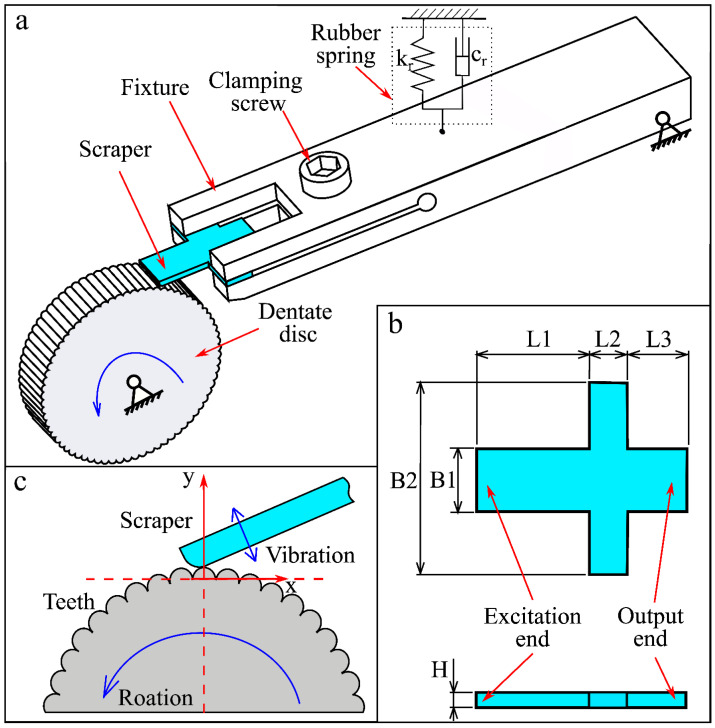
BUV. (**a**) Structure of BUV, (**b**) part drawing of the scraper, (**c**) the scraper and teeth of the dentate disc.

As shown in Figure 2a, this BUV includes a scraper, dentate disc, and fixture system and belongs to a class of mechanical ultrasonic excitation devices. As shown in Figure 2b, the scraper has a cross-shaped structure and is divided into excitation and output ends. As shown in Figure 2a,c, the dentate disc is analogous to the file structure of the leaf-cutting ant, with uniformly distributed micro teeth on its outer cylindrical surface and a tooth count of z_0_. The fixture system included two parts: a rubber spring and a fixture. The fixture clamped the scraper with a clamping screw and was connected to the main frame using a pin to form a rotating pair. Using a rubber spring, a preload force can be applied to the fixture, denoted as F_0_. The fixture system forms a low-frequency mass-spring damping system, and this system can adaptively adjust the spatial position relationship between the dentate disc and the scraper. This fixture system ensures the relative position relationship between the scraper and dentate disc and has a rotational degree of freedom.

During the working process, the dentate disc was rotated uniformly around its central axis at a speed of n_0_. The micro teeth on the dentate disc glide relatively to the scraper, and create an excitation effect on the scraper. The number of teeth passing near the excitation end of the scraper per second is defined as the tooth frequency, denoted by f_z_, which can be calculated using the following formula:

(1) $$f_{z}=\frac{n_{0}\cdot z_{0}}{60}$$

The scraper vibrates under the excitation of the micro teeth. The vibration frequency in the output end of the scraper is defined as the output vibration frequency.

Due to the natural frequency of the mass-spring damping system formed by the fixture system being significantly smaller than the tooth frequency, the fixture remains basically stationary during the working process, thus maintaining a stable fixed effect on the scraper. When the excitation force generated by the teeth was large, the fixture was slowly rotated by a small angle around the pin away from the dentate disc, slowly moving the scraper away from the dentate disc. The distance of the scraper reduces the excitation force of the micro teeth, ultimately achieving a balance between the average excitation force and the pre-force of the rubber spring, and the fixture system enters a stable state. The main function of this fixture system is to maintain stable clamping of the scraper while allowing adaptive fine-tuning of the relative position between the scraper and the dentate disc.

### 2.3. Contact and Engage Relationship between Scraper and Dentate Disc

**Figure 3:**
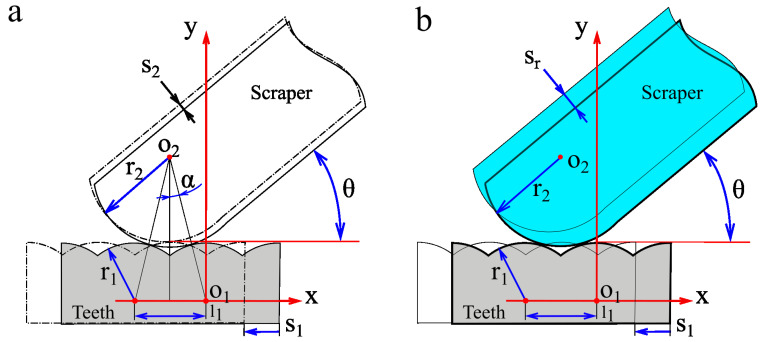
Contact and engage relationship between scraper and dentate disc (**a**,**b**).

The detailed contact relationship between the scraper and the dentate disc is shown in Figure 3. The tooth shape of the dentate disc is a circular arc with radius r_1_ (1 mm) and tooth pitch l_1_ (1 mm). The radius of the scraper tip arc is r_2_ (2 mm). The geometric shapes and size parameters of the teeth and scraper were designed to mimic the excitation organs of leaf-cutting ants. During the device operation, the teeth rotated around the center of the dentate disc. The scraper contacted the teeth and deformed under contact constraints. The scraper periodically vibrates once it passes through the tooth. Assuming that the scraper always maintains contact with the teeth, the geometric relationship between the scraper and teeth is shown in Figure 3a. Under these conditions, the displacement (s_2_) of the scraper can be determined using simple geometric relationships. In actual scenarios, the scraper often cannot maintain continuous contact with the teeth owing to vibrations, as shown in Figure 3b. Therefore, the actual vibration displacement (s_r_) of a scraper is relatively complex and is generally larger than s_2_. The actual vibration displacement of the scraper can only be determined using its dynamic equation.

First, the simple situation shown in Figure 3a was calculated. Owing to the small geometric dimensions of the teeth, the circular arc through which the teeth rotate near the tip of the scraper can be simplified as a straight line, denoted as s_1_. A Cartesian coordinate system was established, with the center of the tooth arc as the origin and the opposite direction of tooth motion in the positive *x*-axis direction. The mechanical factors of the scraper were ignored, and the scraper and dentate disc were assumed to maintain continuous contact. Based on the geometric relationship between the scraper and the dentate disc, the displacement s_2_ of the scraper movement can be calculated.

(2) $$\sqrt[{2}]{{\left(x_{2}-x_{1}\right)}^{2}+{\left(y_{2}-y_{1}\right)}^{2}}=r_{1}+r_{2}$$

Considering the position of the solid line position in Figure 3a as the initial state of the scraper and teeth coordinates o_1_ and o_2_ in the initial state satisfy the following equation:

(3) $$\left\{\begin{array}{c} \left[\begin{array}{c} x_{1}\\ y_{1} \end{array}\right]=\left[\begin{array}{c} 0\\ 0 \end{array}\right]\\ \left[\begin{array}{c} x_{2}\\ y_{2} \end{array}\right]=\left[\begin{array}{c} \left(r_{1}+r_{2}\right)\cdot \cos\left(\theta -\alpha \right)\\ \left(r_{1}+r_{2}\right)\cdot \sin\left(\theta -\alpha \right) \end{array}\right]\\ \alpha =\arcsin\;(\frac{l_{1}}{(r_{1}+r_{2})\cdot 2}) \end{array}\right.$$

During tooth movement, o_1_ and o_2_ satisfy the following equation:

(4) $$\left\{\begin{array}{c} \left[\begin{array}{c} x_{1}\\ y_{1} \end{array}\right]=\left[\begin{array}{c} -s_{1}\cdot \cos\;(\theta )\\ s_{1}\cdot \sin\;(\theta ) \end{array}\right]\\ \left[\begin{array}{c} x_{2}\\ y_{2} \end{array}\right]=\left[\begin{array}{c} \left(r_{1}+r_{2}\right)\cdot \cos\left(\theta -\alpha \right)\\ \left(r_{1}+r_{2}\right)\cdot \sin\left(\theta -\alpha \right)+s_{2} \end{array}\right] \end{array}\right.$$

**Figure 4:**
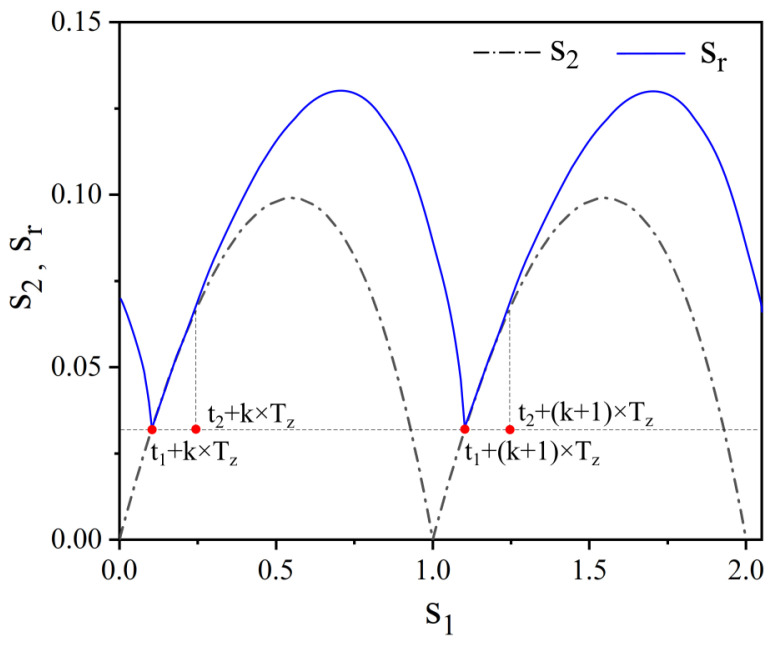
Contact and engage relationship between the scraper and dentate disc.

Combining Equations (2)–(4), s_2_ can be calculated using s_1_, and the graph of s_2_ changing with s_1_ is shown in Figure 4. To illustrate the relationship between s_2_ and s_r_, a possible scenario for s_r_ is plotted in Figure 4. As the tip of the scraper may not always be in contact with the dentate disc, the following relationship holds:

(5) $$s_{r}\geq \;s_{2}$$

As shown in Figure 3b, *T*_z_ is the tooth period and *k* is a positive integer. In a one-tooth period, the start instant of contact is denoted as *t*_1_ + *k* × *T*_z_, and the termination instant of contact is denoted as *t*_2_ + *k* × *T*_z_. During the next tooth period, the start instant of the contact can be denoted as *t*_1_ + (*k +* 1) × *T*_z_, and the termination instant of the contact can be denoted as *t*_2_ + (*k +* 1) × *T*_z_. This was a more probable state, in which the scraper vibrated stably and periodically contacted the dentate disc.

### 2.4. Vibration Model of the Biomimetic Vibrator

**Figure 5:**
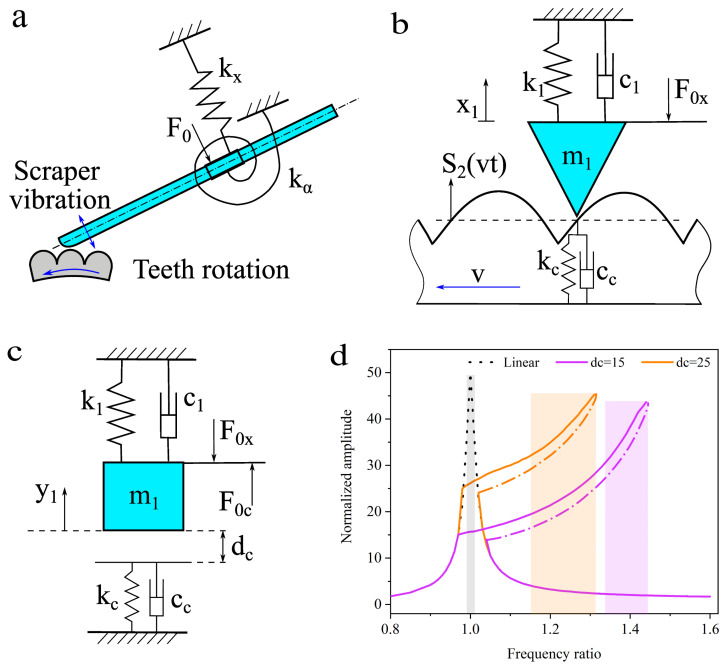
Vibration model. (**a**) A simplified model of the BUV, (**b**) lumped-parameter model, (**c**) equivalent model of lumped-parameter model, (**d**) amplitude–frequency characteristics of the equivalent model.

The scraper vibrates under the excitation of the dentate disc and the fixture forms a constraint on the scraper. This constraint can be simplified as an elastic constraint boundary condition, as illustrated in Figure 5a. To simplify the analysis of the vibration characteristics of the scraper, a lumped-parameter model was established, as shown in Figure 5b. Among them, m_1_ represents the quality characteristics of the scraper material, k_1_ is the comprehensive characteristics of the elasticity and elastic boundary conditions of the scraper material, and c_1_ is the damping characteristics of the scraper material. F_0x_ represents the preload force applied by the fixture on the scraper. Particle m_1_ vibrates under the excitation of the bottom rack, and the tooth shape of the rack is the clenching curve s_2_ between the scraper and the dentate disc. During the vibration process, contact occurs between particle m_1_ and the teeth, and the contact stiffness and damping are denoted as k_c_ and c_c_, respectively.

The clenching displacement s_2_ of the micro teeth conducting on the scraper can be expanded by a Fourier series as follows:

(6) $$s_{2}=a_{0}+\sum_{i=1}^{n}a_{i}\sin\left(i\omega t+\varphi_{i}\right)$$

when $x_{1}\geq s_{2}$, the scraper is not in contact with the micro teeth of the dentate disc, and the scraper forms a free vibration state. When $x_{1}<s_{2}$, the scraper is in contact with the micro teeth of the dentate disc, and the contact force F_cx_ between these two parts can be represented as follows:

(7) $$F_{cx}=\left\{\begin{array}{cc} 0 & x_{1}(t)>s_{2}(t)\\ k_{c}\cdot \left(s_{2}-x_{1}\right)+c_{c}\cdot \left(\dot{s_{2}}-\dot{x_{1}}\right) & x_{1}(t)<s_{2}(t) \end{array}\right.$$

Based on Newton’s second law, the dynamic equation of the vibration system can be represented as

(8) $$m_{1}\ddot{x_{1}}+c_{1}\dot{x_{1}}+k_{1}x_{1}=F_{cx}-F_{0x}$$

Assume $y_{1}=x_{1}-s_{2}$, through a variable substitution method, the dynamic model can be transformed as

(9) $$m_{1}\ddot{y_{1}}+c_{1}\dot{y_{1}}+c_{3}\dot{y_{1}}+k_{1}y_{1}=F_{cy}-F_{0x}+F_{1}$$

where

(10) $$F_{cy}=\left\{\begin{array}{cc} 0 & y_{1}>0\\ k_{c}\cdot \left(-y_{1}\right)+c_{c}\cdot \left(-\dot{y_{1}}\right) & y_{1}<0 \end{array}\right.$$

(11) $$F_{1}=m_{1}\ddot{s_{2}}+c_{1}\dot{s_{2}}+c_{3}\dot{s_{2}}$$

The transformed vibration model is shown in Figure 5c, this is a forced excitation single-degree-of-freedom vibration model with asymmetric piecewise-linear characteristics. This vibration model belongs to a class of nonlinear systems with non-smooth factors that were caused by the periodicity contact between the dentate disc and scraper. Similar single-degree-of-freedom asymmetric piecewise-linear vibration models have been profoundly studied [29,30,31], and researchers have demonstrated that these nonlinear models can generate primary, subharmonic, and superharmonic resonance phenomena. Additionally, the primary resonance frequency and the natural frequency of the linearly derived system are different [30,31].

Based on the above model analysis, it can be concluded that the dentate disc has a dual effect on the vibration of the scraper: providing an excitation force through the geometric relationship between the tooth and scraper, and introducing nonlinear factors into the vibration system through contact relationships between the tooth and scraper.

Ignoring the high-order components of f_cy_, we set the parameter values as shown in Table 1. The Runge–Kutta method was used to solve the vibration model. The amplitude–frequency response curve is shown in Figure 5d.

When the contact factors are ignored, the vibration system degenerates into a single-degree-of-freedom linear vibration model, and its amplitude–frequency response curve is shown by the dashed line in Figure 5d. The operating bandwidth of the degenerate linear vibration model is 0.02. When dc is 25, the operating bandwidth of the nonlinear vibration model is 0.14, which is seven times the bandwidth in the linear state. When dc is 15, the system operating bandwidth of the nonlinear vibration model is 0.08, which is four times the bandwidth in the linear state. Different initial values of dc will form different amplitude–frequency response curves, and different values of dc correspond to different preload forces. Therefore, the preload force of the vibration device will affect the vibration characteristics of the system.

By analyzing the simplified vibration model described above, it can be concluded that biomimetic vibrators comprise a class of strongly nonlinear vibration systems. Nonlinear factors endow vibration systems with broadband vibration characteristics, which are significantly different from those of commonly used piezoelectric and magnetostrictive ultrasonic transducers.

## 3. Experimental Methods and Simulation Model

### 3.1. Experimental Platform

**Figure 6:**
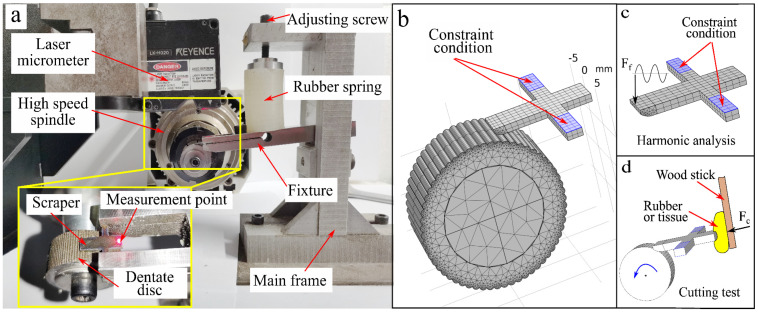
Experimental platform and simulation model. (**a**) Experimental platform, (**b**) finite element model of the BUV, (**c**) finite element model for harmonic analysis of the scraper, (**d**) load-carrying capacity test.

In this study, a prototype scraper was fabricated. A vibration test system comprising a high-speed spindle, wheel-like file, damping spring, and scraper fixture was constructed, as shown in Figure 6a. A wheellike file was installed on a high-speed spindle with an adjustable rotation rate. The scraper was fixed to the fixture and contacted the cylindrical surface of the wheellike file. The high-speed spindle drives the wheel-like file to rotate at a certain speed, and micro teeth on the wheel-like file excite the blade to vibrate. The rotational speed n of the dentate disc was measured using a tachometer. A preload force was applied between the scraper and wheel-like file using a damping spring through the fixture. A laser displacement sensor (LK-H020, Keyence Corp., Osaka, Japan) was used to measure vibration conditions. The vibrations of different areas of the scraper were measured to analyze the vibration conditions.

The material of the dentate disk was 45 steel with a tooth number of 80. The scraper is spring steel 65 Mn, and the specific dimensions of each part of the scraper are listed in Table 2. In the experiment, the scraper was clamped using a fixture and a clamping force was applied through the clamping screws of the fixture. Considering that different clamping forces affect the vibration status of the scraper, all experiments in this article apply a constant torque to the screws using a torque wrench, with a specific torque value of 0.4 Nm, to ensure that the clamping forces of different scraper blades are the same. The tail of the fixture was connected to the main frame of the experimental platform using a hinge to form a rotating pair. In this experiment, a rubber spring was used to apply a preload force to the fixture, creating a predetermined force between the scraper and the teeth. The rubber spring had a stiffness of 100 N/m and a damping coefficient of 1 Ns/m. All experimental rubber springs in this study were subjected to a force of 4 N, and the compression force was set by measuring the compression deformation of the rubber springs. In this experiment, a small amount of lubricating grease was applied to dentate discs.

### 3.2. Experiment for Load-Carrying Capacity

In order to test the load-carrying capacity of the BUV, the load-carrying capacity test was conducted, as shown in Figure 6d. Four test materials, rubber (Ecoflex 00-30), rubber (Ecoflex 00-10), pig muscle tissue, and pig liver tissue, were used in these tests. The muscle and liver tissues of pigs were fresh ex vivo. The test materials were prepared in advance as a rectangular shape of approximately 15 mm × 5 mm × 3 mm. Approximately 1-N force was applied using a wooden strip to press the rubber against the scraper’s output end. The scraper-cutting test was conducted separately with and without ultrasonic vibration, and the results were compared and analyzed.

### 3.3. Simulation Model

We used finite element software (comsol Multiphysics 6.0) to calculate the vibration mode of the scraper. First, based on the size parameters in Table 2, we established a geometric model of the scraper in the 3D modeling software (SOLIDWORKS 2019) and imported it into the finite element software.

According to Table 3, the material parameters were set and the multi-body dynamics module was added as the physical field for calculation. Considering the actual contact situation between the scraper and the fixture, elastic constraints were set on the surface of the contact part between the scraper and the fixture to simulate the actual constraint situation of the scraper in the experiment and ensure computational efficiency. The elastic constraint parameters were obtained through the modal parameter identification method. In the simulation model, the constraint stiffness parameter was set to 3 × 10^7^ N/m, and the damping parameter was set to 1 × 10^2^ s/m. The damping of the scraper material was set using the Rayleigh damping model, *α* = 1522.42, *β* = 1.93 × 10^−7^. The grid was divided as shown in Figure 6b. A modal solver was added to calculate the vibration mode of the scraper. Then, a harmonic excitation force was set at the excitation end, and a frequency domain solver was added to calculate the harmonic response of the scraper output end. In order to investigate the dynamic process of tooth scraping on the blade excitation, a simplified finite element model was established, as shown in Figure 6b. On the basis of modal analysis of the finite element model, we added the dentate disc component. We established a geometric model of the dentate disk based on the corresponding parameters, defined the contact relationship between the scraper and the dentate disc through a penalty function, and set the contact friction coefficient to 0.02. Due to the initial preload force F_0_ of the damping spring on the fixture during the experiment, the scraper underwent elastic deformation. To simplify the calculation model, the initial displacement y_0_ = 0.3 mm of the dentate disc rotation center was set. The rotation speed of the dentate disc was set according to the tooth frequency. The transient solver was added, and the maximum time step was set to 5 µs, so that the calculation time step was less than 10% of the vibration period, to ensure calculation accuracy.

## 4. Results and Discussion

### 4.1. The Operational Frequency Band of the Biomimetic Vibrator

**Figure 7:**
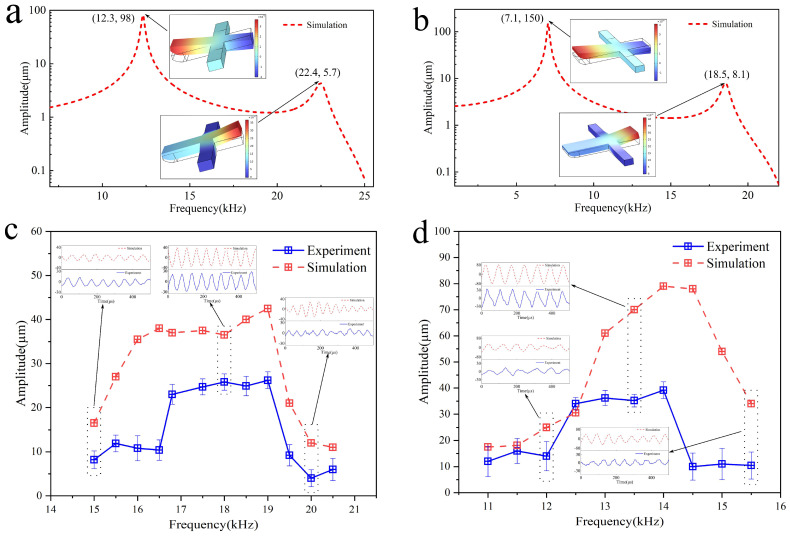
Harmonic response and the operational frequency band. (**a**) Harmonic response of the Sa scraper, (**b**) harmonic response of the Sb scraper, (**c**) operational frequency band of the Sa scraper, (**d**) operational frequency band of the Sb scraper.

Simulation models for the Sa and Sb scraper blades were established, and a harmonic response analysis was conducted. The results are shown in Figure 7a,b. The two peaks of the Sa scraper harmonic response curve correspond to their first and second modes, with modal frequencies of 12.23 kHz and 22.0 kHz, respectively. The first mode can be regarded as the torsional vibration of the main part of the scraper around the support arm, whereas the second mode is more complex. The two peaks of the Sb blade harmonic response curve correspond to their first and second modes, with modal frequencies of 6.23 kHz and 18.0 kHz, respectively. The first mode can be regarded as the torsional vibration of the main part of the scraper around the support arm, whereas the second mode is more complex.

Under different tooth excitation frequencies, finite element calculations and experimental measurements were conducted on the vibration characteristics of the Sa scraper blades. The results are shown in Figure 7c. The finite element calculation results show that stable vibrations can be generated at the output end of the scraper blade when the tooth excitation frequency is in the range of approximately 16–19.2 kHz. The vibration frequency is equal to the tooth excitation frequency, and the vibration amplitude is approximately 35–45 µm. The experimental measurement results show that when the tooth excitation frequency is in the range of approximately 16.8–19 kHz, stable vibration could be generated at the output end of the scraper. The vibration frequency was equal to the tooth excitation frequency, and the vibration amplitude was approximately 22–25 µm. Compared to the simulation results, during the experimental process, the blade vibration frequency bandwidth was smaller, and the vibration amplitude was relatively smaller. This is because the manufacturing error of the tooth plate was ignored in the finite element model; thus, the vibration of the scraper was not affected by the eccentric motion of the tooth plate.

By comparing the vibration phases of each point in the main body of the scraper in the finite element model, it was observed that when the tooth excitation frequency was in the range of approximately 16–19.2 kHz, the main vibration of the scraper was in the first mode. However, the vibration frequency range was higher than its free-mode frequency, which was caused by the nonlinear boundary conditions of the toothed disc, corresponding to the mechanism analysis in Section 3.

Furthermore, finite element calculations and experimental measurements were conducted on the vibration characteristics of the Sb scraper blades at different tooth excitation frequencies. The results are shown in Figure 7d. Finite element calculations show that stable vibrations can be generated at the output end of the scraper blades when the tooth excitation frequency is in the range of approximately 16–19.2 kHz. The vibration frequency is equal to the tooth excitation frequency, and the vibration amplitude is approximately 35–45 µm. The experimental measurement results show that when the tooth excitation frequency is in the range of approximately 16.8–19 kHz, stable vibration could be generated at the output end of the scraper. The vibration frequency was equal to the tooth excitation frequency, and the vibration amplitude was approximately 22–25 µm. Compared to the simulation results, during the experimental process, the blade vibration frequency bandwidth was smaller, and the vibration amplitude was relatively smaller. This is also owing to the neglect of the manufacturing error of the tooth plate in the finite element model, which makes the vibration of the scraper blade unaffected by the eccentric motion of the tooth plate.

By comparing the vibration phases of each point in the main body of the scraper in the finite element model, it was observed that when the tooth excitation frequency was in the range of approximately 16–19.2 kHz, the main vibration of the scraper was in the first mode. However, the vibration frequency range was higher than its free-mode frequency, which was caused by the nonlinear boundary conditions of the toothed disc, corresponding to the mechanism analysis in Section 3. In addition, from the finite element simulation results, it can be seen that compared with the Sa blade, the Sb blade has a larger amplitude change in the range of 16.8–19 kHz, which warrants further in-depth research.

The simulation and experimental results for the Sa and Sb scrapers verified the wideband characteristics of the device. Different sizes of the two scraper blades resulted in different vibration modes and corresponding frequency bands. Therefore, different tooth excitation frequencies must be applied for designs of different sizes. In addition, scraper blades of different sizes can be designed for different frequency vibration requirements. The broadband operating characteristics of this device differ from those of existing piezoelectric and magnetostrictive transducers. When the vibration frequency of the device changes under external loads, the tooth excitation frequency can still be maintained to ensure sufficient vibration energy output. Based on the mechanism analysis in Section 3, it can be concluded that tooth profile clenching between the toothed disc and scraper generates an excitation effect on the scraper. Simultaneously, the contact effect between the toothed disc and scraper introduces nonlinear factors into the vibration system, resulting in broadband vibrations.

### 4.2. Vibration of the Output End

**Figure 8:**
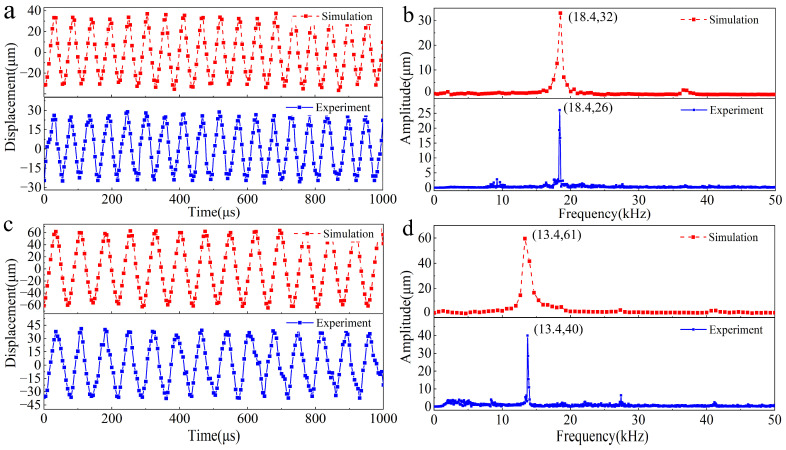
Vibration of the output end. (**a**) Vibration displacement of the Sa scraper, (**b**) vibration spectrum of the Sa scraper, (**c**) vibration displacement of the Sb scraper, (**d**) vibration spectrum of the Sb scraper.

Vibration simulations and experiments were conducted separately on the biomimetic device. In the simulation, set the preload between the toothed disc and the scraper to 35 µm. During the experiment, preloads of 2 N was applied to the damping spring and the vibration displacement of the output end of the scraper was measured. The results are shown in Figure 8. First, simulation calculations and experimental verifications were conducted using a Sa scraper. When the excitation tooth frequency was 18.4 kHz, the Sa scraper generated a stable vibration displacement. The amplitude of the vibration displacement in the simulation results was approximately 32 µm, while in the experimental measurement results, the vibration displacement was approximately 26 µm, as shown in Figure 8a. The experimental results show that the vibration displacement is relatively small, which may be due to the machining error of the gear plate, causing the rotation center to shift. A spectral analysis was performed on the vibration displacement obtained from the simulation and experiment, as shown in Figure 8b. The spectral curve has a distinct main peak corresponding to a frequency of 18.4 kHz, which is equal to the tooth excitation frequency. Except for the main peak, all the other peaks were relatively small, indicating that the vibration displacement was close to a single component of the harmonic vibration.

When the excitation tooth frequency was 13.4 kHz, the Sa scraper generated a stable vibration displacement. The amplitude of the vibration displacement in the simulation results was approximately 61 µm, while in the experimental measurement results, the vibration displacement was approximately 40 µm, as shown in Figure 8c. Similar to the results of Sa scraping, the vibration displacement generated during the experiment was relatively small, which may be due to the machining error of the tooth disk, causing the rotation center to shift. A spectral analysis was performed on the vibration displacement obtained from the simulation and experiment, as shown in Figure 8d. The spectrum curve has an obvious main peak corresponding to a frequency of 13.4 kHz, which is equal to the tooth excitation frequency. Except for the main peak, all the other peaks were relatively small, indicating that the vibration displacement was close to a single component of the harmonic vibration.

The test results for two different blade sizes, Sa and Sb, show that they can generate a stable vibration output, verifying the feasibility of the BUV. This device, which was designed to mimic the vibrating organ structure of leaf-cutting ants, can serve as a new type of excitation device for generating audio- or ultrasound-frequency vibrations. It is a mechanical high-frequency vibration–excitation device.

### 4.3. Vibration of the Fixture

**Figure 9:**
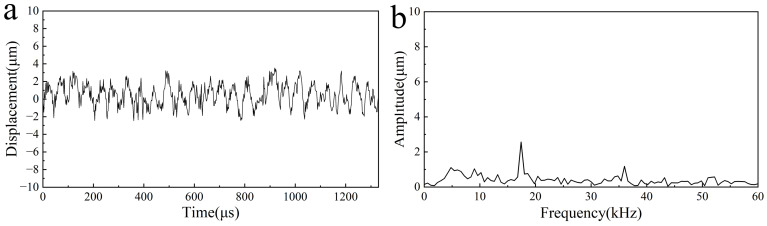
Vibration of the fixture. (**a**) Vibration displacement of the fixture, (**b**) vibration spectrum of the fixture.

The vibration displacement of the fixture was measured and recorded during device operation, as shown in Figure 9a. The vibration displacement amplitude of the fixture is relatively small, approximately 2.8 µm. Compared to the displacement caused by blade vibration, fixture vibration can be ignored. The vibration waveform of the fixture is relatively chaotic, and the corresponding spectrum not only has one main peak but also several other frequency components Figure 9b. The frequency corresponding to the main peak was 18.4 kHz, which is equal to the tooth excitation frequency. The amplitude corresponding to other frequency components outside the main peak is less than 2 µm. Therefore, fixture vibrations can be ignored during the device operation. The experimental results also demonstrated that simplifying the fixture to a fixed one is reasonable. Based on these results, improvements could be made to the fixture and toothed disc parts of the device. This study provides a reference for the design of practical miniature ultrasound surgical scalpels.

### 4.4. Load-Carrying Capacity Test

**Figure 10:**
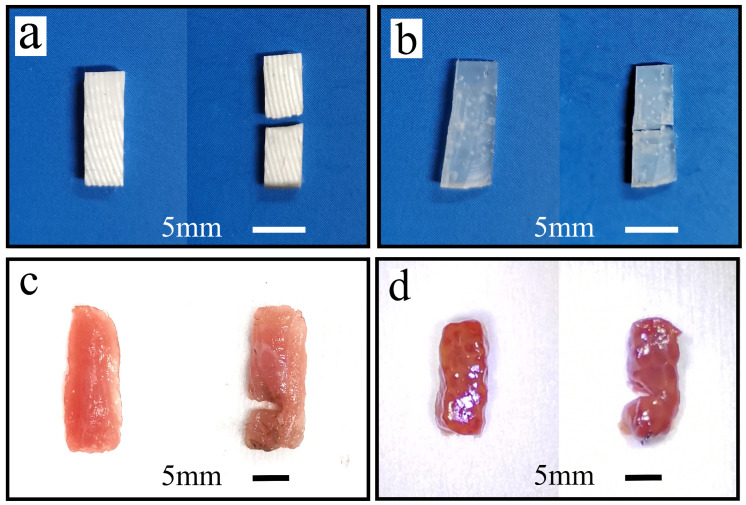
Cutting test of rubber and muscles. (**a**) Rubber (Ecoflex 00-30), (**b**) rubber (Ecoflex 00-10), (**c**) muscle tissues of pigs, (**d**) liver tissues of pigs.

The loading capacity of the designed device was experimentally verified, and Sa scraping blades were used to cut different materials. As shown in Figure 10, the cutting work could be completed for all four materials mentioned above, indicating that the biomimetic device could generate sufficient vibration energy. Simultaneously, the vibration energy output is maintained under the action of the load, indicating that the biomimetic device has a strong load-bearing capacity and is expected to be applied in fields such as minimally invasive surgery.

In this study, two scraper blade sizes were designed for the experimental testing. These results indicate that the biomimetic device can generate stable high-frequency mechanical vibrations. The SA scraper can generate stable vibration output in the range of 17–19 kHz, with a vibration amplitude greater than 25 µm. Cutting experiments have shown that the device can generate sufficient ultrasound power to cut the muscle and liver tissues of pigs. The comprehensive experimental results indicated that the biomimetic device can be used as a new type of power ultrasonic excitation device. This biomimetic device can output sufficient ultrasonic power while also exhibiting broadband operating characteristics that are different from commonly used piezoelectric and magnetostrictive ultrasonic excitation devices.

## 5. Discussion

In this study, we imitated the excitation organs of leaf-cutting ants and designed a BUV. A lump parameter vibration model of the BUV was established, and the vibration model was transformed into a class of forced vibration models with nonsmooth characteristics using the variable substitution method. The teeth of the dentate disk in the vibrator have a dual effect on the scraper: excitation and nonlinear constraints. Using numerical calculation methods, the amplitude–frequency curves of such nonlinear vibration systems were calculated, and it was found that nonlinear factors increased the operating frequency of the vibrator and broadened the operating frequency band.

Subsequently, two scraper sizes were designed for finite element simulation and experimental testing of the vibration characteristics. The results indicate that the device can generate a stable vibration output with a working frequency higher than the first-order natural frequency of the scraper and a wider working frequency band, which is consistent with the theoretical analysis results. In the experiment, the Sa scraper generated a vibration output of approximately 25 µm in the frequency range of 17–19 kHz, indicating that the biomimetic device can be used as a new power ultrasound device. In addition, the Sb blade has a different size design compared to the Sa blade, and experimental results show that the Sb blade can generate high-frequency vibration output in the range of 12.5–14 kHz, with vibration amplitude > 25 µm. Comparative analysis with the Sa scraper shows that blades with different size designs also have different operating frequency ranges. Therefore, for differently sized designs, appropriate tooth excitation frequencies should be set to ensure that the device produces the best working state. In addition, for specific frequency requirements, attempts should be made to design scraper blades of corresponding sizes.

In the simulation model, the vibration output generated by the SA and SB scrapers was higher than that obtained from the experiment, which may be due to a manufacturing error of the tooth disk affecting the vibration effect of the scrapers. Therefore, the working performance of the device could be further improved by increasing the manufacturing accuracy of the gear discs. In the future, we will improve the structural design and manufacturing accuracy of fixtures and toothed discs, and produce practical microultrasonic vibration excitation devices for application in the field of minimally invasive medical robots.

Finally, the loading capacity of the biomimetic vibrator was experimentally tested. The experimental results indicated that the device could complete the cutting of softer rubber and biological tissues. This biomimetic vibrator has sufficient power and load-carrying capacity and is expected to be applied in the fields of microcutting and minimally invasive medicine.

It was discovered that this mechanical vibrator has broadband characteristics and can generate stable ultrasonic vibrations within a wide frequency band. It has sufficient output power and load-carrying capacity within a broadband range to complete the cutting of biological tissues and soft rubber. Overall, compared with piezoelectric and magnetostrictive ultrasonic transducers, a broader operating frequency band and the potential to produce more miniature ultrasound scalpels are two advantages of the BUV. In the future, this device will be optimized and designed based on the findings of this study. A miniature mechanical power ultrasound vibrator will be developed, and animal experiments will be conducted to test the feasibility of using the biomimetic vibrator as a minimally invasive surgical instrument.

## 6. Conclusions

A biomimetic ultrasonic vibrator was developed by observing and characterizing the vibration organs of leaf-cutting ants.

First, the BUV can operate in a frequency range of 16.8–19 kHz. Within the working frequency range, the vibration of the BUV was stable and the amplitude of the vibration displacement was higher than 22 µm. The BUV can serve as a new type of power ultrasonic excitation device.

Second, the operating frequency band of the BUV was approximately 2.2 kHz which is broader than those of the piezoelectric and magnetostrictive ultrasonic transducers. This phenomenon is due to the nonlinear factor caused by the periodic contact between the scraper and dentate disc.

Finally, the BUV was able to cut soft rubber and pig tissues. These results indicated that the output power and load-carrying capacity of the BUV were sufficient for its use as an ultrasonic scalpel. This study provides a promising new method for producing miniature ultrasound scalpels that can be used in minimally invasive surgeries.

## Figures and Tables

**Table 1:** Nondimensionalized parameters.

Symbol	Value
m_1_	1
k_1_	1
kc	8 × k_1_
c_1_	0.01 × 2 × (k_1_ × m_1_)^0.5^
c_c_	0.01 × 2 × (k_c_ × m_1_)^0.5^

**Table 2:** Size parameters of scrapers (unit: mm).

Number	L1	L2	L3	B1	B2	H
S-1	4.5	1.6	2.8	8.0	20.0	1.0
S-2	7	1.6	3.6	8.0	20.0	1.0

**Table 3:** Material parameters.

Material	Density	Young’s Modulus	Poisson’s Ratio
ASTM-1045 [32]	7850 Kg/m^3^	209 GPa	0.26
ASTM-1566 [32]	7810 Kg/m^3^	197 GPa	0.25

## Data Availability

The datasets generated during and/or analyzed during the current study are available from the corresponding author on reasonable request.
